# Enchondromas and atypical cartilaginous tumors at the proximal humerus treated with intralesional resection and bone cement filling with or without osteosynthesis: retrospective analysis of 42 cases with 6 years mean follow-up

**DOI:** 10.1186/s12957-018-1437-z

**Published:** 2018-07-13

**Authors:** Georg W. Omlor, Vera Lohnherr, Jessica Lange, Simone Gantz, Christian Merle, Joerg Fellenberg, Patric Raiss, Burkhard Lehner

**Affiliations:** Center of Orthopaedics, Trauma Surgery and Paraplegiology, Heidelberg University Hospital, Schlierbacher Landstrasse 200a, 69118 Heidelberg, Germany

**Keywords:** Enchondroma, Atypical cartilaginous tumor, Chondrosarcoma, Humerus, Curettage, Osteosynthesis

## Abstract

**Background:**

Enchondromas and atypical cartilaginous tumors (ACT) are often located at the proximal humerus. Most lesions can be followed conservatively, but surgical resection may alleviate pain, avoid pathological fractures, and prevent transformation into higher grade chondrosarcomas. Rigorous intralesional resection and filling with polymethylmethacrylate bone cement has been proposed for enchondromas but also for ACT, as an alternative for extralesional resection. We intended to analyze radiological, clinical, and functional outcome of this strategy and compare bone cement without osteosynthesis to bone cement compound osteosynthesis, which has not been analyzed so far.

**Methods:**

We retrospectively analyzed 42 consecutive patients (mean follow-up 73 months; range 8–224) after curettage and bone cement filling with or without osteosynthesis. Exclusion criteria were Ollier’s disease and cancellous bone filling. Twenty-five patients only received bone cement. Seventeen patients received additional proximal humerus plate for compound osteosynthesis to increase stability after curettage. Demographics and radiological and clinical outcome were analyzed including surgery time, blood loss, hospitalization, recurrences, and complications. An additional telephone interview at the final follow-up assessed postoperative satisfaction, pain, and function in the quick disabilities of the arm, shoulder, and hand (DASH) score and the Musculoskeletal Tumor Society (MSTS) score. Statistics included the Student *T* tests, Mann-Whitney *U* tests, and chi-square tests.

**Results:**

No osteosynthesis compared to compound osteosynthesis showed smaller tumors (4.2 (± 1.5) cm versus 6.6 (± 3.0) cm; *p* = 0.005) and smaller bone cement fillings after curettage (5.7 (± 2.1) cm versus 9.6 (± 3.2) cm; *p* = 0.0001). A score evaluating preoperative scalloping and soft-tissue extension did not significantly differ (1.9 (± 0.9) versus 2.0 (± 1.0); rating scale 0–4; *p* = 0.7). Both groups showed high satisfaction (9.2 (± 1.5) versus 9.2 (± 0.9); *p* = 0.5) and low pain (1.0(±1.7) versus 1.9(±1.8); *p* = 0.1) in a rating scale from 0 to 10. Clinical and functional outcome was excellent for both groups in the DASH score (6.0 (± 11.8) versus 11.0 (± 13.2); rating scale 0–100; *p* = 0.2) and the MSTS score (29.0 (± 1.7) versus 28.7 (± 1.1); rating scale 0–30; *p* = 0.3). One enchondroma recurrence was found in the group without osteosynthesis. Complications (one fracture and one intra-articular screw) were only detected after osteosynthesis. Osteosynthesis had longer surgery time (70 (± 21) min versus 127 (± 22) min; *p* < 0.0001), more blood loss (220 (± 130) ml versus 460 (± 210) ml; *p* < 0.0001), and longer stay in the hospital (6 (± 2) days versus 8 (± 2) days; *p* = 0.004).

**Conclusions:**

Intralesional tumor resection was oncologically safe and clinically successful with or without osteosynthesis. Osteosynthesis did not reduce the risk for fracture but was more invasive.

## Background

Chondrogenic tumors such as enchondromas and atypical cartilaginous tumors (ACT = chondrosarcoma grade I according to older nomenclature) are often located at the proximal humerus [1] and raise the question for surgical therapy. Enchondromas at the shoulder are often found incidentally on radiographs or after imaging due to unspecific pain but differentiation between more frequently found enchondromas and rather rarely found ACT only by pain seems impossible [1, 2]. Clinical, radiological, and even histological differentiation between enchondromas and the more aggressive low-grade malignant ACT is extremely challenging [3–5]. In case of clinically inactive lesions, conservative treatment with clinical and radiological follow-up seems sufficient [6]. In case of clinical and radiological aggressiveness with endosteal scalloping, soft-tissue extension, lesion growth or size > 6 cm, and pain not related to other shoulder co-morbidities, most authors prefer surgical treatment [1, 7–9]. Intralesional resection is well accepted for benign enchondromas where less aggressive lesions may also be filled biologically with cancellous bone [10] whereas bone cement filling has the advantage of reduced recurrence rates due to heat destruction of potentially remaining tumor cells, which may be beneficial for large and radiologically aggressive enchondromas, where differentiation from ACT is difficult [1, 9, 11]. The gold standard treatment for ACT with highest oncological safety is extralesional resection [12, 13], but recent studies report sufficient oncological safety and significantly better clinical and functional results after intralesional curettage of ACT located in the long bones [1, 7–9, 11, 14–21]. Intralesional therapy, however, seems not appropriate for ACT of the pelvis and trunk as well as for cases with local recurrence of ACT indicating a more aggressive phenotype [22].

Due to lack of evidence in the literature, we intended to retrospectively analyze our large series of 42 consecutive patients with enchondroma or ACT of the proximal humerus surgically treated with rigorous intralesional excision and bone cement filling either with additional proximal locking compression plate (LCP) humerus plate or without a plate. Whether additional osteosynthesis as a bone cement compound osteosynthesis [23–25] is beneficial at the proximal humerus has not been analyzed so far, so we compared both groups regarding the following research questions: Is clinical and functional outcome different? Is radiologic appearance of the lesions different? Is there a difference in recurrences, complications, or other surgical parameter?

## Methods

**Fig. 1:**
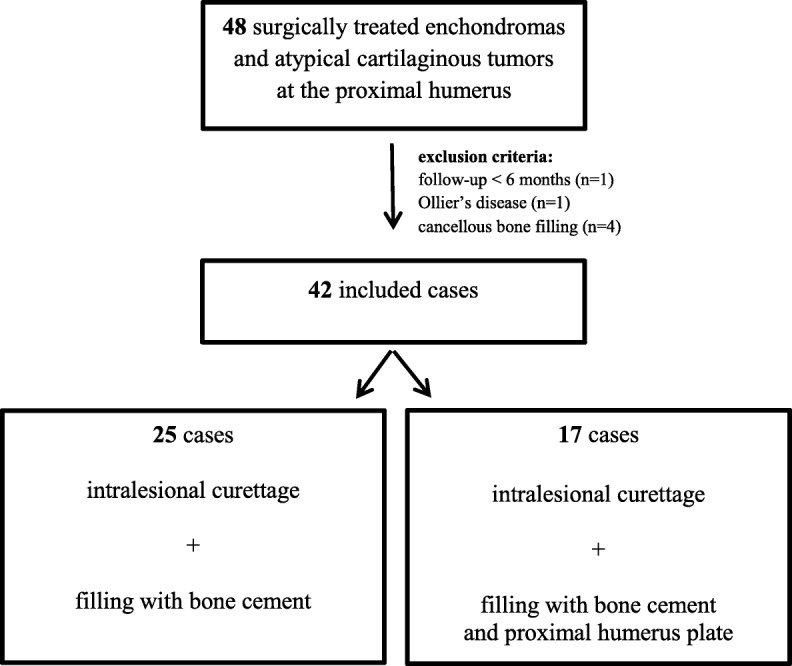
Flowchart of inclusion criteria

**Fig. 2:**
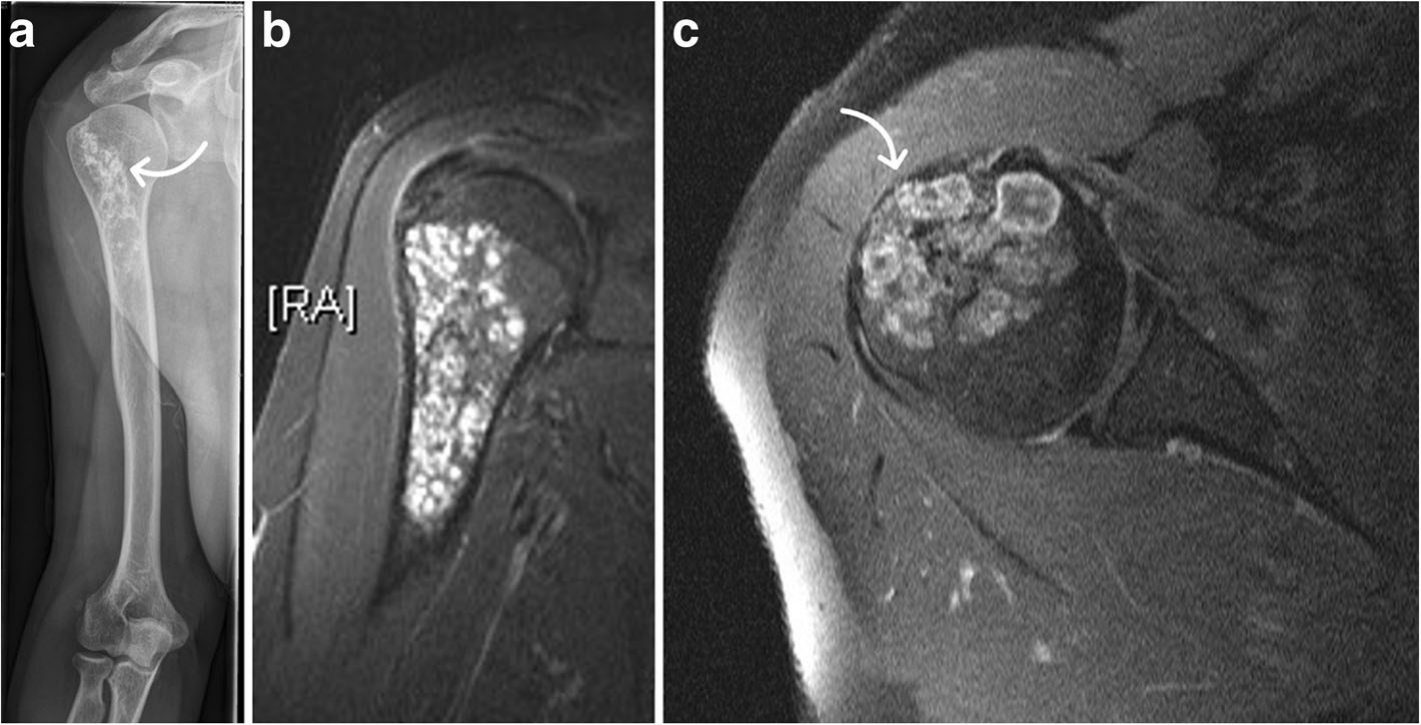
Painful large chondrogenic tumor at the proximal humerus prior to surgery without osteosynthesis. Plain radiograph (**a**) shows typical popcorn-like calcifications (arrow) inside the epiphyseal and metaphyseal lesion. STIR MRI sagittal series (**b**) and T1-weighted contrast-enhanced axial MRI series (**c**) reveal large size and aggressiveness with endosteal scalloping (arrow) reducing stability of the proximal humerus

**Fig. 3:**
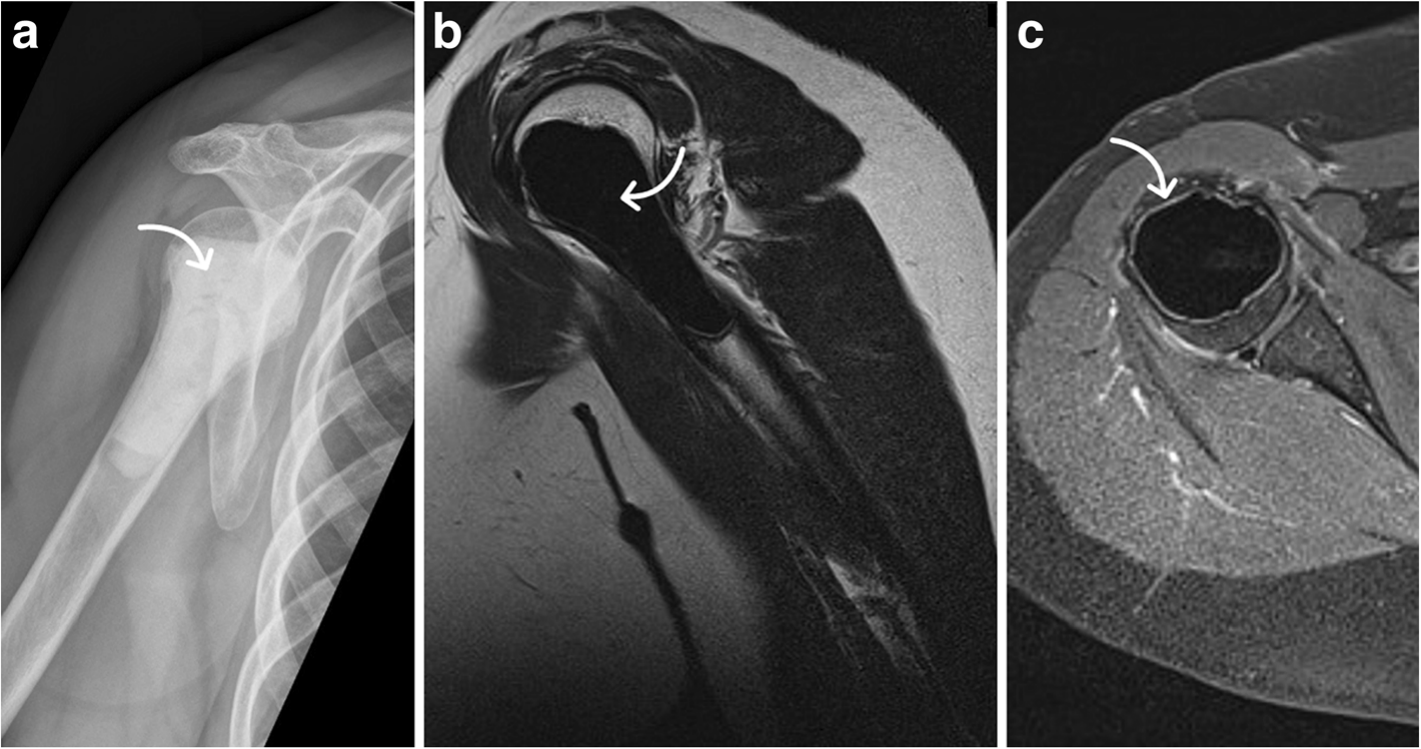
Bone cement filling without osteosynthesis after rigorous intralesional tumor resection. The large bone cement filling (arrow) can be easily depicted in plain radiographs (**a**) and MRI with T2-weighted sagittal series (**b**) and T1-weighted contrast-enhanced axial series (**c**). Although filling out the complete proximal humerus, sufficient stability was achieved without additional osteosynthesis as no postoperative fractures were found later on for all equally treated cases. Typical edema line (arrow in **c**) after bone cement implantation must be distinguished from local recurrence

**Fig. 4:**
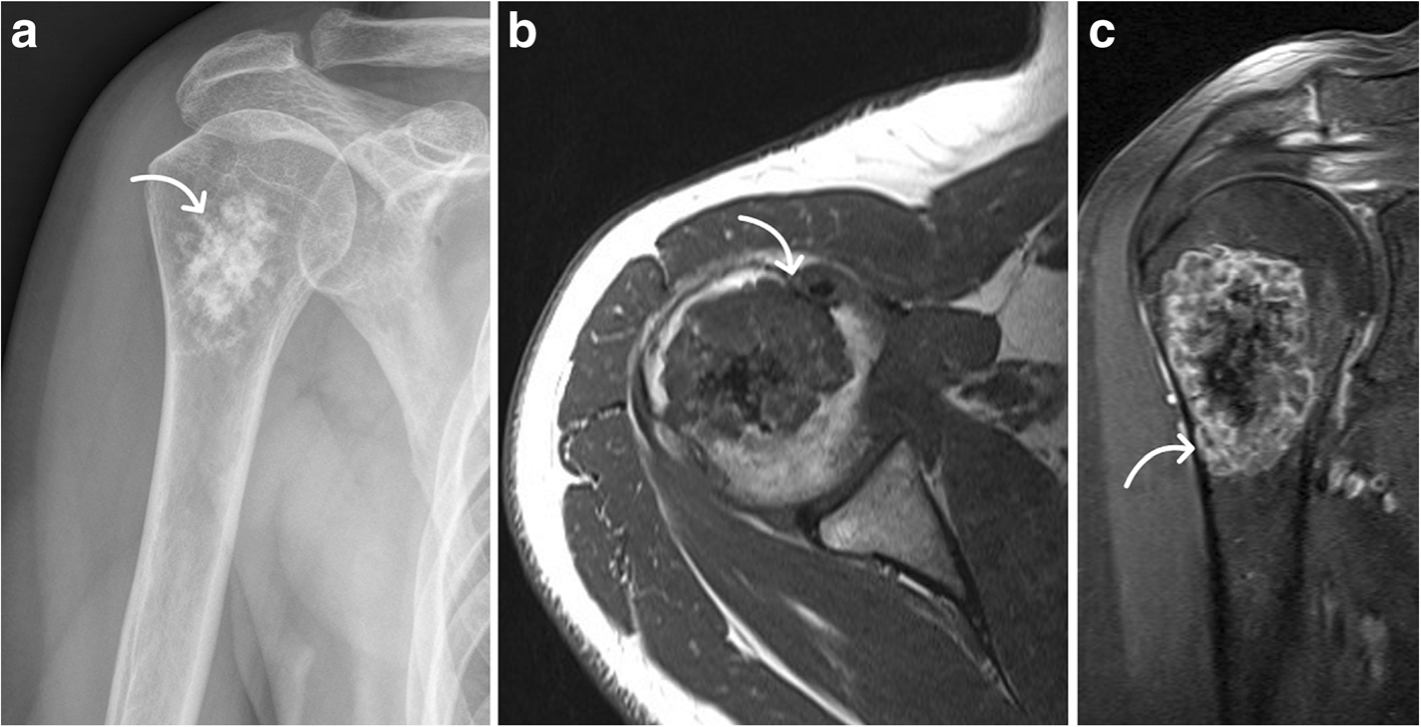
Painful large chondrogenic tumor at the proximal humerus prior to surgery with additional osteosynthesis. Plain radiograph (**a**) with typical popcorn-like calcifications (arrow) and T1-weighted axial MRI series (**b**) and T1-weighted contrast-enhanced coronal series (**c**) with endosteal scalloping (arrows) and reduced stability without significant differences compared to lesions treated without osteosynthesis (compare Fig. 1)

**Fig. 5:**
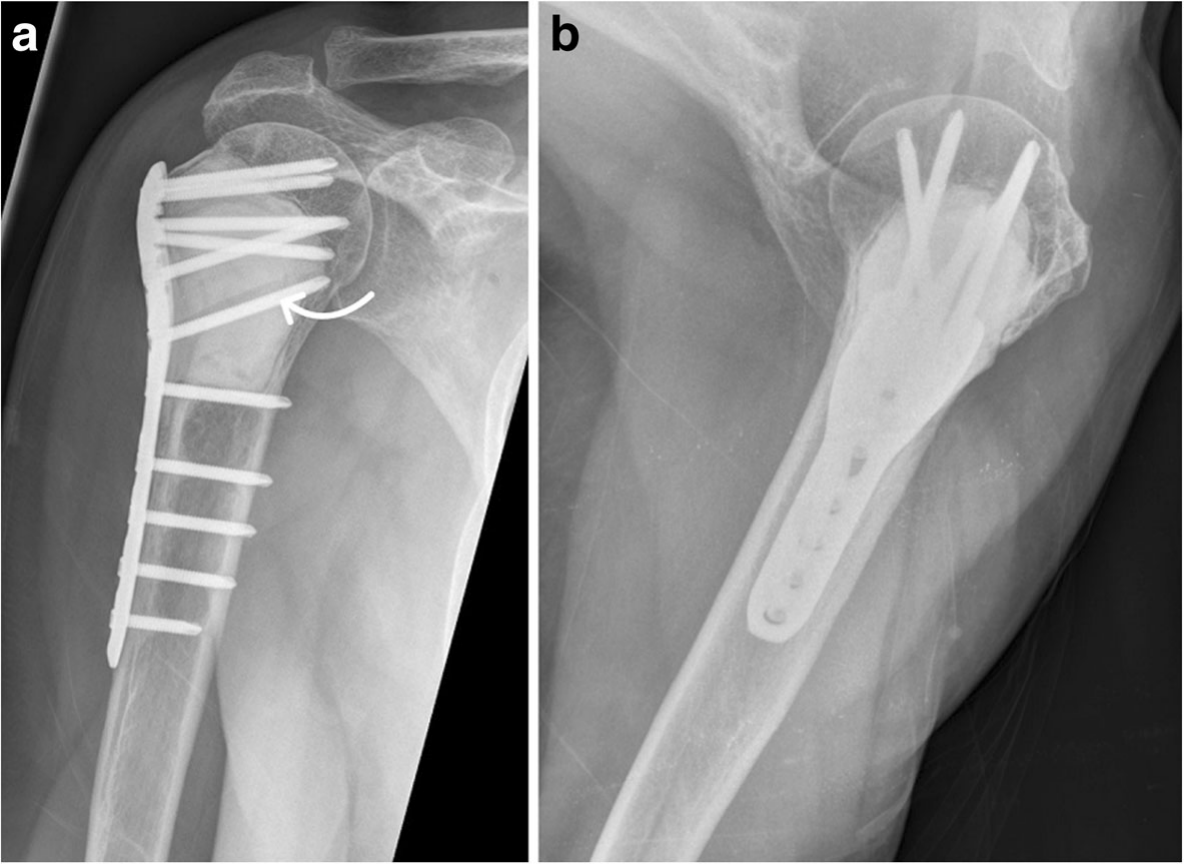
Bone cement filling with additional compound plate osteosynthesis after rigorous intralesional tumor resection. Plain radiographs in ap (**a**) and axial (**b**) view show additional stabilization with a proximal locking compression plate of the humerus with integration of the screws inside the bone cement (arrow) to potentially increase stability of the proximal humerus

We retrospectively analyzed 42 consecutive patients surgically treated for enchondroma or ACT at the proximal humerus with a mean follow-up of 73 months (range 8–224). Approval was given by our local ethical committee. From 2005 till 2017, we found a total of 113 patients treated conservatively or surgically at our orthopedic oncology outpatient clinic (level I bone and soft-tissue tumor center and orthopedic and trauma surgery university hospital). Exclusion criteria were no surgical therapy (*n* = 65), less than 6 months follow-up (*n* = 1), Ollier’s disease (*n* = 1), and filling with cancellous bone (*n* = 4) instead of bone cement, as cancellous bone filling was only used for smaller and less aggressive enchondromas and hence could not be compared. Forty-two patients with sufficient data which were surgically treated between 2006 and 2016 were finally included in the study (Fig. 1). Of those, 25 patients underwent rigorous intralesional excision with use of a high-speed burr and filling of the lesion with polymethylmethacrylate bone cement (Palacos® R+G; Heraeus Medical, Hanau, Germany) to achieve improved stability and reduce recurrence rate due to heat destruction of potentially remaining tumor cells during the polymerization process in the lesion cave (Figs. 2 and 3). This group was defined as the study group. Seventeen other patients underwent the same procedure followed by the support of a proximal humerus locking compression plate (LCP; PHILOS plate, Synthes GmbH, Oberdorf, Switzerland) in a way that the screws of the osteosynthesis were integrated into the bone cement as a compound plate osteosynthesis (Figs. 4 and 5). This group was defined as the control group.

A decision whether additional compound plate osteosynthesis with a proximal humerus plate was used or not was done individually. According to the documented medical records, additional plate osteosynthesis was justified and selected, if preoperative imaging and intraoperative appearance after curettage caused doubt of sufficient stability only with bone cement. As there are no scientific guidelines for decision-making, we retrospectively analyzed the potential criteria.

Adopted from other studies [1], lesion size was measured by the maximal diameter in MRI, as heterogeneous geometrical configurations of the lesions hamper valid measurement of lesion volumes. Preoperative lesion size and postoperative size of the cavity filled with bone cement after curettage were evaluated. Preoperative aggressiveness of the lesion was judged by a semi-quantitative score considering soft-tissue extension (no = 0, yes = 1) and endosteal scalloping (no = 0, minimal = 1, moderate = 2, high = 3) resulting in a score from 0 to 4. Scalloping was considered minimal if it involved less than one third of the cortical thickness, moderate if it involved up to two thirds, and high if it involved more than two thirds. Radiological evaluation with x-rays and MRIs was performed initially and regularly with intervals between 6 (first year after surgery) and 12 months for clinical routine. Imaging was evaluated together with our musculoskeletal radiologists subspecialized in bone and soft-tissue tumor diagnostic.

Patient demographics (Table 1) and clinical histories including detailed information on surgical treatment, histological analysis, recurrences, and complications were analyzed. For systematic evaluation of pain, patient satisfaction, and functional outcome at final follow-up, we performed an additional telephone interview. Remaining pain and overall patient satisfaction were asked to be judged from 0 to 10. Limitations and clinical function were evaluated by the Musculoskeletal Tumor Society (MSTS) score [26] and the quick disabilities of the arm, shoulder, and hand (DASH) score [27].

**Table 1:** Demographics of both treatment groups

	Intralesional resection + bone cement (*n* = 25)	Intralesional resection + bone cement + proximal humerus plate (*n* = 17)	Statistical test with *p* value
Gender			
Male	*n* = 4	*n* = 8	Chi-square test *p* = 0.03
Female	*n* = 21	*n* = 9	
Age			
Mean (± SD)	50.3 (± 10.8) years	48.2 (± 12.0) years	Mann-Whitney *U* test *p* = 0.52
Histology			
Enchondroma	*n* = 19	*n* = 12	Chi-square test *p* = 0.67
ACT	*n* = 3	*n* = 2	
Enchondroma or ACT	*n* = 3	*n* = 3	

To evaluate the results, statistical analysis was performed for the outcome measures “MSTS score”, “DASH score”, “preoperative lesion size”, “scalloping and soft-tissue extension score”, “size of bone filling”, “number of recurrences”, “number of complications”, “blood loss”, “surgery time”, and “days of hospitalization”. To compare the differences, Student *T* tests, Mann-Whitney *U* tests, and chi-square tests were calculated depending on the scale level and distribution of the data. Statistical significance was assumed at a *p* value < 0.05. Due to the exploratory character of the study, all *p* values are interpreted descriptively. Analysis was performed together with the statistician of our department (SG) using SPSS for Windows 22.0 (SPSS Inc., USA).

## Results

All 42 patients were followed until the final follow-up with information on surgeries, radiological outcome, clinical presentation in the outpatient clinic, recurrences, and complications. Results from the telephone interview were only available for *n* = 31 patients.

### Surgical parameter

The group without additional osteosynthesis showed less surgery time, less intraoperative blood loss, and less days of hospitalization (Table 2).

**Table 2:** Surgical parameter

	Intralesional resection + bone cement (*n* = 25)	Intralesional resection + bone cement + proximal humerus plate (*n* = 17)	Statistical test with *p* value
Surgery time			
Mean (± SD)	70 (± 21) min	127 (± 22) min	Mann-Whitney *U* test *p* < 0.0001
Blood loss			
Mean (± SD)	220 (± 130) ml	460 (± 210) ml	Mann-Whitney *U* test *p* < 0.0001
Days of hospitalization			
Mean (± SD)	6 (± 2) days	8 (± 2) days	Mann-Whitney *U* test *p* = 0.004

### Radiological outcome

Lesions which did not receive additional osteosynthesis were smaller (4.2 (± 1.5) cm versus 6.6 (± 3.0) cm; *p* = 0.005). After curettage, the lesion cavity which was filled with bone cement was also smaller in the group without osteosynthesis (5.7 (± 2.1) cm versus 9.6 (± 3.2) cm; *p* = 0.0001). Endosteal scalloping in the group without osteosynthesis was minimal in 10 cases, moderate in 10 cases, and high in 5 cases. In the group with osteosynthesis, it was minimal in five cases, moderate in seven cases, and high in four cases. Soft-tissue extension was only found in four cases (two in each group). A semi-quantitative score of preoperative scalloping and soft-tissue extension did not show significant differences between the groups (1.9 (± 0.9) versus 2.0 (± 1.0); rating scale 0–4; *p* = 0.7). Detailed results depending on histological diagnosis are presented in Table 3.

**Table 3:** Radiological outcome

	Intralesional resection + bone cement (*n* = 25)	Intralesional resection + bone cement + proximal humerus plate (*n* = 17)	Statistical test with *p* value
Initial tumor size			
Mean (± SD)			Student *T* test
All	4.2 (± 1.5) cm	6.6 (± 3.0) cm	*p* = 0.005
Enchondroma	4.2 (± 1.7) cm	7.6 (± 3.0) cm	*p* = 0.003
ACT	3.7 (± 1.2) cm	5.3 (± 1.1) cm	*p* = 0.24
Enchondroma or ACT	4.3 (± 0.6) cm	4.5 (± 2.9) cm	*p* = 0.26
Cavity size after curettage			
Mean (± SD)			Student *T* test
All	5.7 (± 2.1) cm	9.6 (± 3.2) cm	*p* = 0.0001
Enchondroma	5.7 (± 2.2) cm	10.2 (± 2.5) cm	*p* < 0.0001
ACT	4.7 (± 1.1) cm	5.6 (± 0.4) cm	*p* = 0.44
Enchondroma or ACT	6.9 (± 0.3) cm	9.0 (± 4.6) cm	*p* = 0.65
Scalloping + soft-tissue extension score			
Mean (± SD) rating scale 0–4			Mann-Whitney *U* test
All	1.9 (± 0.9)	2.0 (± 1.0)	*p* = 0.71
Enchondroma	1.8 (± 1.0)	1.8 (± 0.9)	*p* = 0.81
ACT	2.3 (± 0.6)	3.0 (± 1.4)	*p* = 0.52
Enchondroma or ACT	2.0 (± 1.0)	2.0 (± 1.0)	*p* = 1.0

### Clinical outcome

Overall clinical outcome was excellent. Patient satisfaction, pain, and functional outcome did not show statistically significant differences. Detailed results are presented in Table 4.

**Table 4:** Clinical and functional outcome

	Intralesional resection + bone cement (*n* = 19)	Intralesional resection + bone cement + proximal humerus plate (*n* = 12)	Statistical test with *p* value
Satisfaction			
Mean (± SD) rating scale 0–10	9.2 (± 1.5)	9.2 (± 0.9)	Mann-Whitney *U* test *p* = 0.5
Pain			
Mean (± SD) rating scale 0–10	1.0 (± 1.7)	1.9 (± 1.8)	Mann-Whitney *U* test *p* = 0.1
DASH score			
Mean (± SD) rating scale 0–100	6.0 (± 11.8)	11.0 (± 13.2)	Mann-Whitney *U* test *p* = 0.2
MSTS score			
Mean (± SD) rating scale 0–30	29.0 (± 1.7)	28.7 (± 1.1)	Mann-Whitney *U* test *p* = 0.3

### Recurrences

No recurrence was found in the osteosynthesis group. One of the 25 patients from the group without osteosynthesis had a histologically proven enchondroma recurrence after 4 years. It was successfully treated by revision surgery, again without osteosynthesis. Difference in recurrence was not significant (chi-square test *p* = 0.4).

### Complications

No complications were found in the group without osteosynthesis. In the osteosynthesis group, two of the 17 patients had complications. This difference did not reach statistical significance but showed a trend (chi-square test *p* = 0.08). As complications, we found one peri-implant fracture which was, however, related to a fall with adequate trauma. It was treated by re-osteosynthesis with a longer plate. Another patient needed revision surgery due to a postoperatively found intra-articular screw.

## Discussion

Intralesional resection with vigorous curettage and filling the cavity with bone cement have been described in several studies to treat enchondromas and low-grade malignant ACT resulting in sufficient oncological safety and excellent function [1, 7–9, 11, 14–21]. Stability after curettage is an important issue, as postoperative fractures are frequently described [10, 13, 17–19]. Biomechanically demanding locations such as the femur are at highest risk [10, 19], but fractures also occur in the upper extremity including the proximal humerus [13, 18, 23]. Bone cement compound osteosynthesis may increase stability [23, 24], but it remains unclear whether this approach is beneficial at the proximal humerus or not [25]. Advantages and disadvantages have not been analyzed so far, so we for the first time compared intralesional tumor resection and bone cement filling with and without additional plate osteosynthesis at the proximal humerus.

Preoperative tumor size and size of the tumor cavity after curettage were significantly different with smaller lesions in patients without osteosynthesis and larger lesions in patients with additional osteosynthesis. Hence, surgeons will have to judge larger lesions to be at higher risk for instability or postoperative fracture and therefore decide to implant additional osteosynthesis more often in these cases. All lesions were radiologically judged as aggressive, without significant difference of the groups in the scalloping and soft-tissue extension score. Consequently, size will have predominantly influenced the decision towards osteosynthesis. Besides generally high radiologic aggressiveness of the lesions of the present series, only 11 lesions were histologically diagnosed as ACT or potential ACT compared to the majority of histologically benign diagnosed enchondromas. Valid differentiation between ACT and aggressive enchondromas by histology might be questionable. This is supported by several other studies, documenting difficult or even impossible differentiation of both entities [1, 3–5].

In case of additional osteosynthesis, surgery time was significantly longer with significantly more blood loss and longer hospital stay afterwards. Hence, it would be beneficial to avoid additional osteosynthesis. Significant clinical and functional differences regarding MSTS score, DASH score, pain, and satisfaction were not found after intralesional resection with or without osteosynthesis. Both treatment groups had excellent clinical outcome with high satisfaction, low pain, and only minimal functional impairments. Compared to other studies on intralesional resection strategy, our MSTS score results were similar and even slightly better [1, 9, 11, 13, 16].

As reported by others, this strategy not only maintains excellent function but also offers sufficient oncological safety [1, 7–9, 11, 14–21]. In our series, no ACT recurrence but one enchondroma recurrence was found 4 years after intralesional tumor resection with bone cement filling without osteosynthesis. Patients with additional osteosynthesis did not show recurrence. Higher recurrence rate in cases without additional osteosynthesis might theoretically be explained by less radical tumor resection, as the surgeon might have been afraid of instability. Difference in recurrence was not significant but valid comparison is not possible due to only one found recurrence. As a disadvantage of additional osteosynthesis, postoperative MRI images showed higher artifacts although the plates are made of titanium. This problem, however, can be sufficiently solved with the latest MRI technology using artifact suppressing algorithms [28], so local tumor recurrences can still be ruled out with sufficient reliability. We did not find pulmonary metastases until the final follow-up. ACT as a grade I malignancy and even enchondromas offer potential risk for transformation into higher grade chondrosarcomas which has been reported with percentages from 1 to 9% [5, 6, 29]. The highest risk is known for tumors of the axial skeleton, pelvis, and truck; hence, intralesional therapy seems less appropriate in these cases [1, 12, 13, 30]. In case of ACT recurrence, a more aggressive phenotype is expected with higher risk for transformation into higher grade chondrosarcoma, so most authors recommend wide resection in such cases [22, 30]. The lowest risk is expected for ACT of the long bones of the appendicular skeleton without statistical evidence for differences between the upper and lower extremity [30]. Nevertheless, the literature more often reports on recurrences at the femur and tibia [1, 13, 20] compared to the humerus [11]. In the series of Andreou et al. including 225 patients with ACT, 46 lesions were in the upper extremity but no transformation into higher grade chondrosarcoma or pulmonary metastases were found contrary to 5 transformations into grade II chondrosarcoma with additional pulmonary metastases in ACT located in the femur and tibia. Analysis of metastasis-free survival, however, was not significantly different although the overall number of analyzed cases was very high in this multicenter study [30].

We had two complications, and they were only found after additional osteosynthesis. The intra-articular screw can be directly related to the procedure. The postoperative fracture had an adequate trauma and hence cannot be clearly attributed to the osteosynthesis. In case of additional osteosynthesis, superior stability would be expected, but our data cannot prove this. No fractures were found after intralesional excision without additional osteosynthesis, but one fracture was found although osteosynthesis was added. This can be interpreted differently. First, the theoretical stability increase of additional osteosynthesis might be overestimated. Second, surgeons might have used additional osteosynthesis more often than needed, to achieve the highest safety. The literature does not give sufficient answers on whether additional osteosynthesis should be used or not. A series including 10 humerus cases treated with intralesional resection and bone cement filling without osteosynthesis reported no fractures [9]. Dierselhuis et al. found 11 fractures in 108 cases but there were only 33 humerus cases, of those 2 had fractures, and detailed information on prior osteosynthesis is not given [18]. Kim et al. analyzed 36 cases and found 4 fractures only located at the femur whereas no fracture was found for the 23 humerus lesions although additional osteosynthesis was not used [19]. In our series, the largest cavity treated without osteosynthesis was 12.4 cm compared to 15.2 cm for the largest cavity in the osteosynthesis group. As we did not find fractures in the group without osteosynthesis, we can only conclude that our mean cavity size of 5.7 (± 2.1) cm was in a safe zone at the proximal humerus and that even larger sizes up to 12.4 cm did not show problems without osteosynthesis. For better interpretation, higher patient numbers would be beneficial and further biomechanical cadaver studies should be performed to achieve objective data considering primary stability of bone cement fillings with and without additional osteosynthesis at the proximal humerus. So far, no data is available in the literature.

Conservative follow-up without surgery might be an important alternative [6], although psychological and socioeconomic aspects of leaving an aggressive tumor inside the body with need for long-time radiologic follow-up have to be considered [31]. Conservative strategy is not further discussed here, as the goal of the present study was comparison of two surgical strategies.

Several limitations have to be mentioned. Due to the retrospective study design, pain and function were not systematically evaluated preoperatively, so postoperative clinical success could not be compared to the preoperative situation. Furthermore, decision for or against additional osteosynthesis was done individually without a standardized decision protocol with a potential selection bias.

## Conclusion

Our series documents oncologically safe and clinically successful outcome no matter if intralesional tumor resection was performed with bone cement filling alone or with additional osteosynthesis at the proximal humerus. Compound plate osteosynthesis with the intention to increase stability did not reveal significant clinical disadvantages besides longer surgery time, more blood loss, and longer hospitalization. Further biomechanical evaluations and randomized studies should be initiated.

## Abbreviations

ACT: Atypical cartilaginous tumor (chondrosarcoma grade I according to older WHO classification)
DASH score: Quick disabilities of the arm, shoulder, and hand score
LCP: Locking compression plate
MSTS score: Musculoskeletal Tumor Society score
